# Dynamically remodeled hepatic extracellular matrix predicts prognosis of early-stage cirrhosis

**DOI:** 10.1038/s41419-021-03443-y

**Published:** 2021-02-08

**Authors:** Yuexin Wu, Yuyan Cao, Keren Xu, Yue Zhu, Yuemei Qiao, Yanjun Wu, Jianfeng Chen, Chen Li, Rong Zeng, Gaoxiang Ge

**Affiliations:** 1 State Key Laboratory of Cell Biology, Shanghai Institute of Biochemistry and Cell Biology, Center for Excellence in Molecular Cell Science, Chinese Academy of Sciences, 200031 Shanghai, China; 2 University of Chinese Academy of Sciences, 100049 Beijing, China; 3 CAS Key Laboratory of Systems Biology, Shanghai Institute of Biochemistry and Cell Biology, Center for Excellence in Molecular Cell Science, Chinese Academy of Sciences, 200031 Shanghai, China; 4 School of Life Science, Hangzhou Institute for Advanced Study, University of Chinese Academy of Sciences, 310024 Hangzhou, China; 5 Center for Single-Cell Omics, School of Public Health, Shanghai Jiao Tong University School of Medicine, 200025 Shanghai, China; 6 CAS Key Laboratory of Systems Biology, Hangzhou Institute for Advanced Study, University of Chinese Academy of Sciences, 310024 Hangzhou, China; 7 School of Life Science and Technology, Shanghai Tech University, 201210 Shanghai, China

**Keywords:** Proteomics, Prognostic markers, Liver fibrosis

## Abstract

Liver cirrhosis remains major health problem. Despite the progress in diagnosis of asymptomatic early-stage cirrhosis, prognostic biomarkers are needed to identify cirrhotic patients at high risk developing advanced stage disease. Liver cirrhosis is the result of deregulated wound healing and is featured by aberrant extracellular matrix (ECM) remodeling. However, it is not comprehensively understood how ECM is dynamically remodeled in the progressive development of liver cirrhosis. It is yet unknown whether ECM signature is of predictive value in determining prognosis of early-stage liver cirrhosis. In this study, we systematically analyzed proteomics of decellularized hepatic matrix and identified four unique clusters of ECM proteins at tissue damage/inflammation, transitional ECM remodeling or fibrogenesis stage in carbon tetrachloride-induced liver fibrosis. In particular, basement membrane (BM) was heavily deposited at the fibrogenesis stage. BM component minor type IV collagen α5 chain expression was increased in activated hepatic stellate cells. Knockout of minor type IV collagen α5 chain ameliorated liver fibrosis by hampering hepatic stellate cell activation and promoting hepatocyte proliferation. ECM signatures were differentially enriched in the biopsies of good and poor prognosis early-stage liver cirrhosis patients. Clusters of ECM proteins responsible for homeostatic remodeling and tissue fibrogenesis, as well as basement membrane signature were significantly associated with disease progression and patient survival. In particular, a 14-gene signature consisting of basement membrane proteins is potent in predicting disease progression and patient survival. Thus, the ECM signatures are potential prognostic biomarkers to identify cirrhotic patients at high risk developing advanced stage disease.

Liver cirrhosis remains major health problem^1,2^. Nearly all chronic liver diseases eventually lead to liver fibrosis and cirrhosis^1,2^. Fibrotic liver diseases predispose to liver failure, portal hypertension, and are associated with increased risk of liver cancer^1,2^. Serum biomarkers and liver stiffness measurement are becoming increasingly useful in liver cirrhosis diagnosis, in particular asymptomatic early-stage cirrhosis^3^. However, prognostic biomarkers are needed to identify cirrhotic patients at high risk developing advanced stage disease. Child-Pugh score, determined by scoring five clinical measures, including total bilirubin, serum albumin, prothrombin time, presence of ascites and hepatic encephalopathy, is used to assess the prognosis of chronic liver disease, in particular cirrhosis^4^. Child-Pugh Class A liver cirrhosis patients have the least severe liver disease and the highest survival rate, compared to the Class B (moderately severe liver disease) or Class C (most severe liver disease) patients^5,6^. A 186-gene signature has been shown to predict clinical outcomes of patients with hepatitis C-related Class A early-stage cirrhosis^5^. Despite these progresses, there is still urgent need for robust and sensitive prognostic biomarkers for patients with liver cirrhosis, especially early-stage liver cirrhosis.

Liver fibrosis is part of the general wound healing response to many causes of chronic injury^7,8^. Chronic injury causes sustained inflammation, myofibroblast activation and aberrant extracellular matrix (ECM) deposition. Bioinformatic analysis of proteins containing characteristic domains commonly found in ECM proteins, as well as proteomic analyses of proteins in purified ECM have identified ~300 core ECM proteins, including ~200 glycoproteins, 43 collagens, and 36 proteoglycans (http://matrisomeproject.mit.edu)^9,10^. In addition, there are large numbers of ECM-associated proteins, including ECM-affiliated proteins, which share domain similarity with core ECM proteins or are known to be associated with ECM proteins, ECM regulators that are responsible for regulating ECM remodeling, and secreted factors bound to core ECM proteins^9,10^. Core ECM proteins and ECM-associated proteins are collectively known as matrisome^9,10^. Core ECM proteins and ECM-associated proteins cooperatively assemble and remodel ECM and regulate cellular functions through cell surface receptors. Compositions of matrisome are qualitatively and quantitatively different in normal and diseased tissues^10–18^. Such ECM changes are highly dynamic during wound healing and tissue fibrosis. In skin wound healing, types XII and XIV collagens and tenascin C are found throughout skin wound healing, while latent TGF-β binding protein-4 is detected in the later phase of wound repair^11^. Fibronectin assembles into fibrillar structures that provide scaffold for collagen fibril assembly. Deposition of fibronectin is an early event in both wound healing and tissue fibrosis^19,20^. Blocking fibronectin deposition decreases collagen accumulation and improves liver function during liver fibrogenesis^21^. Ethanol and/or acute lipopolysaccharide challenge induce transitional ECM remodeling and dramatically increase the number of hepatic ECM proteins before fibrotic changes are evident in the liver^12^.

We thus hypothesize that dynamically remodeled hepatic matrix may possess prognostic value for liver fibrosis and cirrhosis. Here, we used enriched hepatic matrix and proteomic approach to study the dynamic changes of the ECM during the progression of experimental liver fibrosis. Unique matrisomal proteins were identified for tissue damage/inflammation, transitional ECM remodeling and fibrogenesis. Such matrisomal protein signatures, including basement membrane signature, are predictive of Child-Pugh Class A early-stage cirrhosis progression and patient survival. A 14-gene signature, present in both cluster 4 and basement membrane signatures, predicts progression and patient survival of Child-Pugh Class A early-stage cirrhosis.

## Results

### Global changes of the hepatic matrisomal proteins in liver fibrosis

**Fig. 1:**
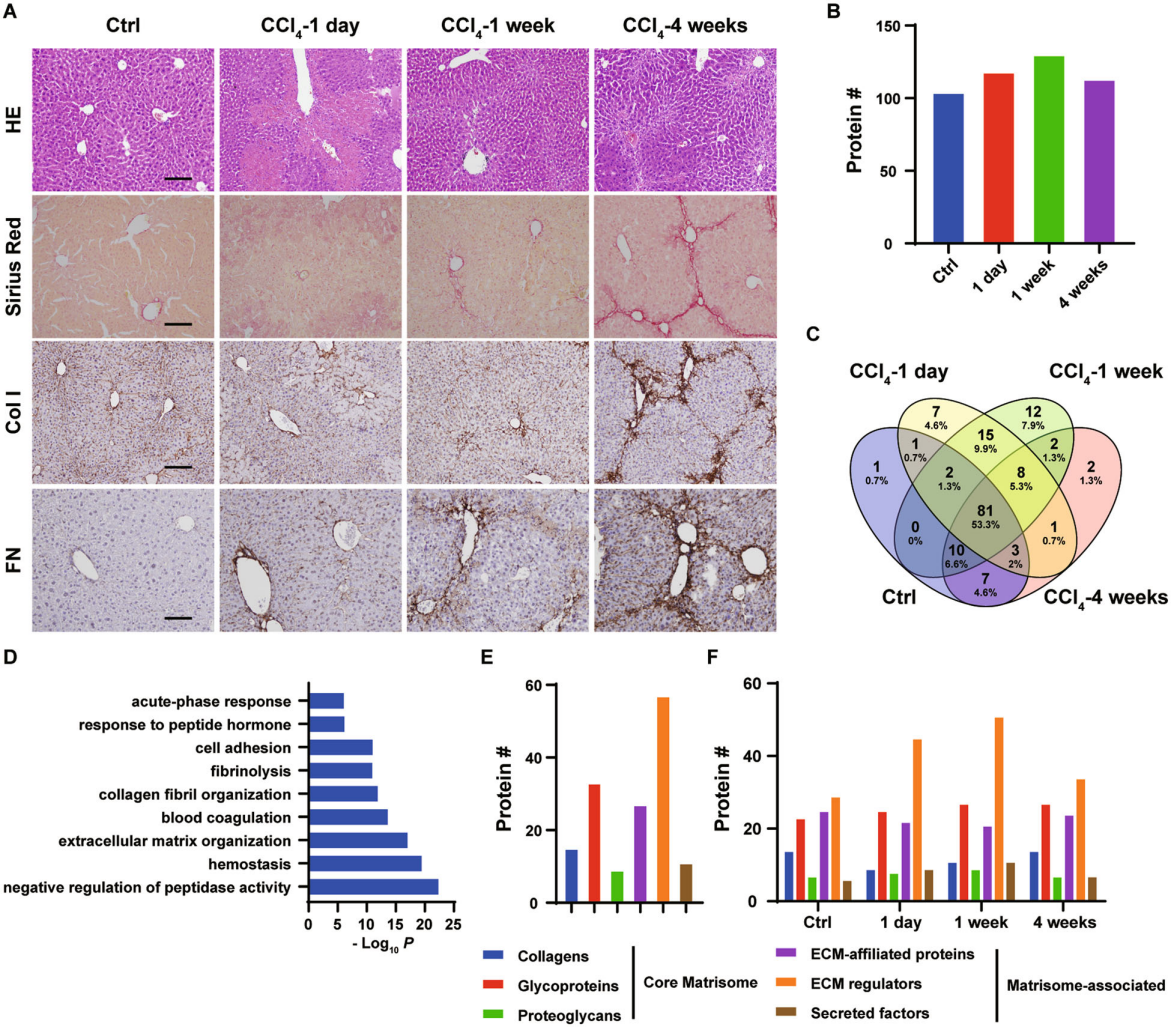
Identification of hepatic matrisome composition in CCl_4_-induced liver fibrosis model. **A** Microscopic views of livers from mice intraperitoneally injected with CCl_4_ for 1 day, 1 week or 4 weeks or olive oil for 4 weeks. Liver sections were stained with hematoxylin-eosin (HE), Picrosirius Red or immunostained for type I collagen (Col I) or fibronectin (FN). Scale bars: 100 μm. **B**–**E** Decellularized and enriched hepatic matrices were subjected to mass-spec analyses. **B** Numbers of matrisomal proteins identified at each liver fibrogenesis stage. **C** Venn diagram depicting overlap of matrisomal proteins across four liver fibrogenesis stages. **D** Gene ontology (GO) of identified matrisomal proteins. **E** Numbers of identified proteins in matrisomal classes. **F** Numbers of identified proteins in matrisomal classes at each liver fibrogenesis stage.

Repeated carbon tetrachloride (CCl_4_) treatment leads to iterative wound healing, resulting in deregulated ECM deposition and progressive development of liver fibrosis^22,23^. At 1-day after CCl_4_ treatment, there were large areas of hepatocyte death and inflammation, as well as large number of proliferating cells (Fig. 1A and S1). At 1-week after CCl_4_ treatment, hepatic necrosis was not evident, and the inflammation and cell proliferation ceased (Fig. 1A and S1). Weak collagen deposition was observed after 1-week CCl_4_ treatment (Fig. 1A). 4-week repeated CCl_4_ treatment resulted in extensive collagen deposition and α-smooth muscle actin (αSMA)-positive myofibroblast accumulation (Fig. 1A and S1). Deposition of fibronectin is an early event in tissue fibrosis that provides scaffold for collagen fibril assembly^19,20^. Fibronectin was deposited as early as 1-day after CCl_4_ treatment and heavily deposited in the fibrotic livers after 4-week CCl_4_ treatment (Fig. 1A). 1-day, 1-week and 4-week CCl_4_ treatment thus represented stages of tissue damage/inflammation, transitional ECM remodeling and fibrogenesis.

During the development of liver fibrosis, ECM undergoes dynamic remodeling. Decellularization by non-ionic detergents removes soluble intracellular and extracellular proteins, while preserves insoluble ECM scaffold and proteins tightly bound to the ECM scaffold^24^. Hepatic matrices were enriched and subjected to in-depth proteomic analyses. Proline and lysine residues are heavily hydroxylated in collagens and other ECM proteins^10,25,26^. To estimate the abundance of matrisomal proteins more accurately, both proline and lysine hydroxylation was included in database search^13^. A total of 152 matrisomal proteins were identified, in which 81 matrisomal proteins were identified in all 4 groups (Figs. 1B, C and Table S1). Each group yielded a distinct ECM protein profile (Fig. 1C and Table S1). In particular, 7 (LAMA1, LGALSL, MBL, CTSG, MASP1, SERPINB1A, SERPINB9C) and 12 (MFGE8, ASPN, CST3, F10, HYAL1, MMP19, P4HA2, SERPINA1C, SERPINA1D, SERPIND1, SERPINF1, NGF) matrisomal proteins were only identified in the 1-day and 1-week treatment groups, respectively (Fig. 1C and Table S1). Additional 15 matrisomal proteins were identified in both 1-day and 1-week treatment groups (CRELD2, IGFBP1, TSKU, PRG4, CSTB, F12, HABP2, SERPINA1B, SERPINA1E, SERPINA3N, SERPINA7, SERPINF2, ANGPTL4, CRLF3, S100A8), but not in the control and 4-week groups (Fig. 1C and Table S1), suggesting the transitional nature at these two time points. COL15A1 and P4HA1 were only identified in the 4-week treatment group (Fig. 1C and Table S1). Gene Ontology (GO) analysis revealed that these matrisomal proteins are involved in negative regulation of peptidase activity and ECM organization (Fig. 1D).

### Differential changes of the matrisome subgroups in liver fibrosis

The matrisome comprises structural ECM proteins, termed as core ECM, as well as ECM-associated proteins^10^. A total of 57 core ECM proteins (15 collagens, 33 glycoproteins and 9 proteoglycans) and 95 ECM-associated proteins (27 ECM-affiliated proteins, 57 ECM regulators and 11 secreted factors) were identified (Fig. 1E). The numbers of identified glycoproteins, proteoglycans, ECM-affiliated proteins and secreted factors were comparable among different groups (Fig. 1F, Table S1). However, the numbers of identified collagens and ECM regulators drastically changed after 1-day and 1-week CCl_4_ treatment (Fig. 1F, Table S1). Less collagen proteins were identified (9 and 11 vs. 14 in control and 4-week groups), while more ECM regulators were detected (45 and 51 vs. 29 and 34 in control and 4-week groups) after 1-day and 1-week CCl_4_ treatment (Fig. 1F, Table S1). Gene set enrichment analysis (GSEA)^27^ revealed dynamic changes of core matrisome and matrisome-associated proteins during CCl_4_-induced liver fibrosis (Fig. S2). Compared to the control group, significantly downregulated core matrisome protein^10^ expression and upregulated matrisome-associated protein^10^ expression was detected after 1-day CCl_4_ treatment (Fig. S2). Consistent to the prominent liver fibrosis, core matrisome was significantly enriched, whereas matrisome-associated protein expression was significantly decreased after 4-week CCl_4_ treatment (Fig. S2). The subgroups of matrisomal proteins were differentially regulated during CCl_4_-induced liver fibrosis. Both collagens^10^ and proteoglycans^10^ were quantitatively downregulated after 1-day treatment, compared to the control group (Fig. S3A and S3C). Collagen expression was only restored after 4-week, but not 1-week CCl_4_ treatment (Fig. S3A). Unlike the collagens, expression of proteoglycans^10^ was partially restored in 1-week group that was further increased in 4-week group (Fig. S3C). Expression of ECM regulators^10^ was significantly upregulated in 1-day group, compared to the control group that was further upregulated after 1-week treatment (Fig. S3E). Expression of ECM regulators^10^ was sharply decreased after 4-week treatment (Fig. S3E). Expression of glycoproteins, ECM-affiliated protein and secreted factors^10^ only moderately changed during CCl_4_-induced liver fibrosis (Fig. S3B, S3D and S3F).

### Dynamic changes of the matrisome in liver fibrosis

**Fig. 2:**
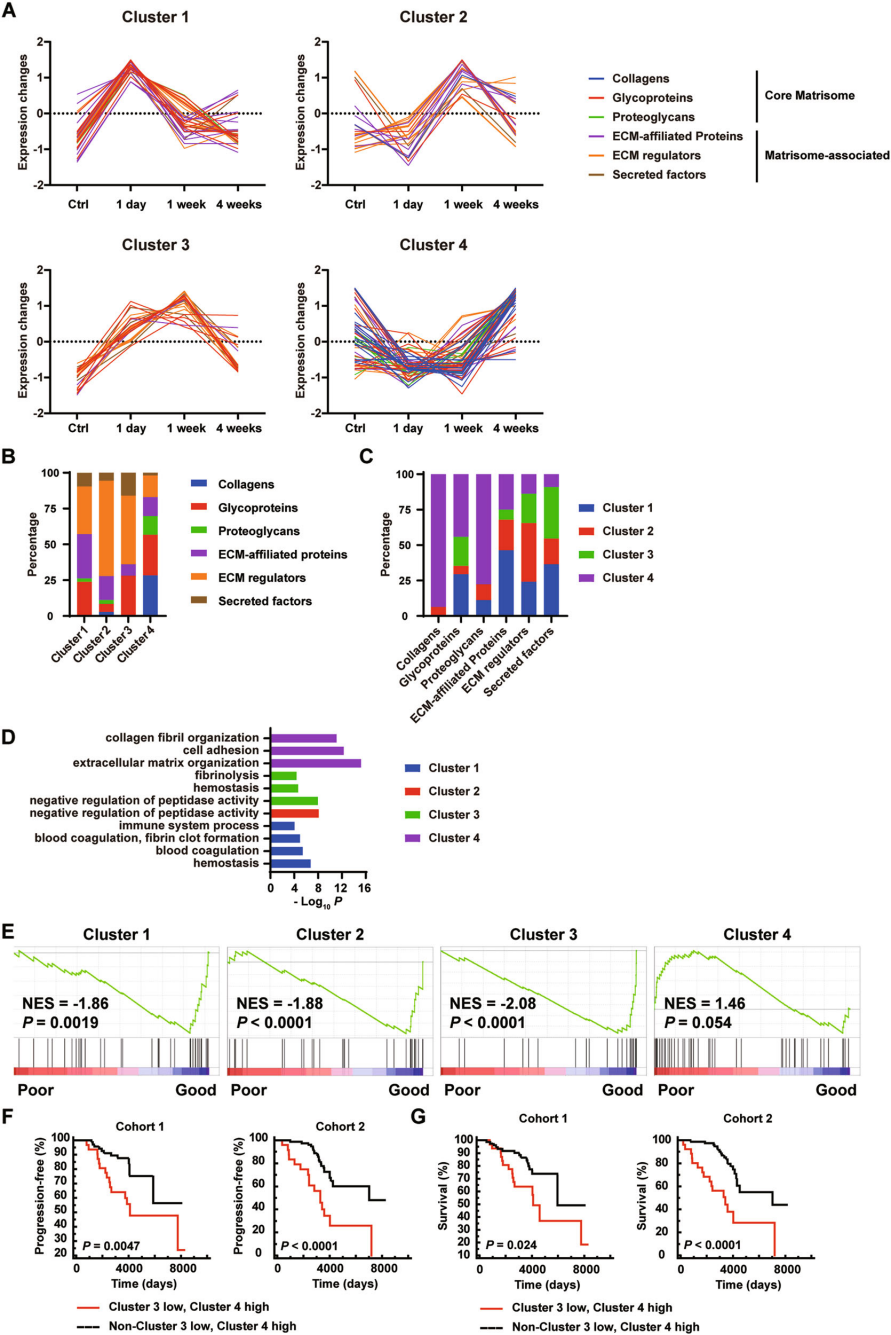
Hepatic proteins in matrisomal classes are dynamically remodeled in CCl_4_-induced liver fibrosis model, and predict disease progression and survival of early-stage liver cirrhosis patients. **A** Clustered profiles of protein abundances for identified matrisomal proteins. **B** Distribution of matrisomal classes in each cluster. **C** Distribution of clusters in each matrisomal class. **D** Gene ontology (GO) of each cluster. **E** GSEA analyses comparing enrichment of ECM clusters between good prognosis and poor prognosis Child-Pugh Class A early-stage liver cirrhosis biopsies. **F**, **G** Probabilities of disease progression from Child-Pugh Class A to Class B or C (**F**) and survival (**G**) of early-stage cirrhosis patients according to the expression levels of the clusters-3 and cluster-4 ECM signatures.

Matrisomal proteins changed in relative abundance in response to CCl_4_ treatment. Clustering analysis of matrisomal protein quantitation identified four clusters (Fig. 2A–C and Table S2). Cluster 1 represented matrisomal proteins upregulated at 1-day after CCl_4_ treatment, containing mainly glycoproteins, ECM-affiliated proteins, and ECM regulators. Cluster 2 represented matrisomal proteins upregulated at 1-week after CCl_4_ treatment, containing mainly ECM regulators and ECM-affiliated proteins. Cluster 3 represented matrisomal proteins upregulated at 1-day and 1-week after CCl_4_ treatment, containing mainly ECM regulators and glycoproteins. Cluster 4 represented matrisomal proteins decreased at 1-day or 1-week, but restored at 4-week after CCl_4_ treatment, containing mainly collagens, glycoproteins, and ECM regulators. Consistent to the histological changes of acute tissue damage/inflammation at 1-day, transitional ECM remodeling at 1-week, and fibrogenesis at 4-week after CCl_4_ treatment (Fig. 1A), GO analysis revealed each cluster was associated with distinct biological processes (Fig. 2D). Cluster 1 contained proteins involved in the regulation of tissue damage and immune responses. Cluster 2 and 3 contained proteins involved in the negative regulation of peptidase activity and ECM remodeling. Cluster 4 contained proteins that are involved in the regulation of collagen fibril and ECM organization. Glycoproteins fibrinogen chains (FGA, FGB, FGG) and secreted factors S100A8/S100A9^28^ responsible for blood clotting and active inflammatory response were highly expressed at 1-day, but expression of these glycoproteins sharply decreased at 1-week after CCl_4_ treatment (Fig. S4). Expression of laminin and periostin, glycoproteins required for liver fibrosis development^29^, decreased at 1-day and 1-week, but restored at 4-week after CCl_4_ treatment (Fig. S4). Type V collagen is a low abundance fibrillar collagen incorporated into type I collagen fibrils to regulate the size and shape of the type I collagen fibrils^30^. Similar to that of type I collagen, expression of type V collagen decreased at 1-day, but increased at 4-week after CCl_4_ treatment (Fig. S4).

Cirrhosis represents the terminal stage of chronic fibrotic liver diseases. In addition to clinical prognostic variables, e. g., platelet count and serum bilirubin levels, 186-gene signature was used to predict outcomes of patients with hepatitis C-related Child-Pugh Class A early-stage cirrhosis, and classify the patients as having poor and good prognosis of disease progression to Class B or C advanced stage disease and patient death^5^. Expression of cluster-1, cluster-2, or cluster-3 matrisomal proteins were significantly enriched in the liver biopsies from the Class A early-stage cirrhosis patients least likely developing Class B or Class C advanced stage disease (good prognosis group), compared to that from the patients at high risk developing Class B or Class C advanced stage disease (poor prognosis group) (Fig. 2E and S5A). In contrast, cluster-4 matrisomal proteins were enriched in the poor prognosis group (Fig. 2E and S5A). The differential enrichment of each cluster indicated that dynamically remodeled ECM is actively involved in disease progression. We next sought to determine whether the clinical outcomes of the cirrhotic patients could be predicted by expression of the ECM clusters in the Child-Pugh Class A early-stage liver cirrhosis samples^5^. Cox analyses indicated that clusters-3 and-4 were predictive for the progression of Child-Pugh class and patient survival (Tables S3). To avoid potential heterogeneity among the samples, we randomly split 216 samples into two data sets with similar size (107 and 109 cases) and similar clinicopathologic characteristics (Table S4). Patients with low cluster-3 and high cluster-4 matrisomal protein expression had significantly shorter disease progression-free survival time than the rest of the patients (low cluster-3/low cluster-4, high cluster-3/low cluster-4, or high cluster-3/high cluster-4 matrisomal protein expression) in both Cohort-1 and Cohort-2 (Cohort-1: HR: 3.29 (1.44–7.54), *P* = 0.0047; Cohort-2: HR: 6.06 (2.59–14.19), *P* < 0.0001) (Fig. 2F). Ten-year rates of Child-Pugh class progression were 44% vs. 12% (Cohort-1) and 65% vs. 27% (Cohort-2) for patients with or without low Cluster 3/high cluster 4 matrisomal protein expression pattern (Fig. 2F). Patients with low cluster-3/high cluster-4 matrisomal protein expression had significantly shorter overall survival time than the patients without low cluster-3/high cluster-4 matrisomal protein expression pattern (Cohort-1: HR: 2.46 (1.13–5.39), *P* = 0.024; Cohort-2: HR: 8.26 (3.38–20.20), *P* < 0.0001) (Fig. 2G). Ten-year survival rates were 64% vs. 84% (Cohort-1) and 38% vs. 80% (Cohort-2) for patients with or without low cluster-3/high cluster-4 matrisomal protein expression pattern (Fig. 2G).

### Basement membrane proteins are prognostic for early-stage liver cirrhosis

**Fig. 3:**
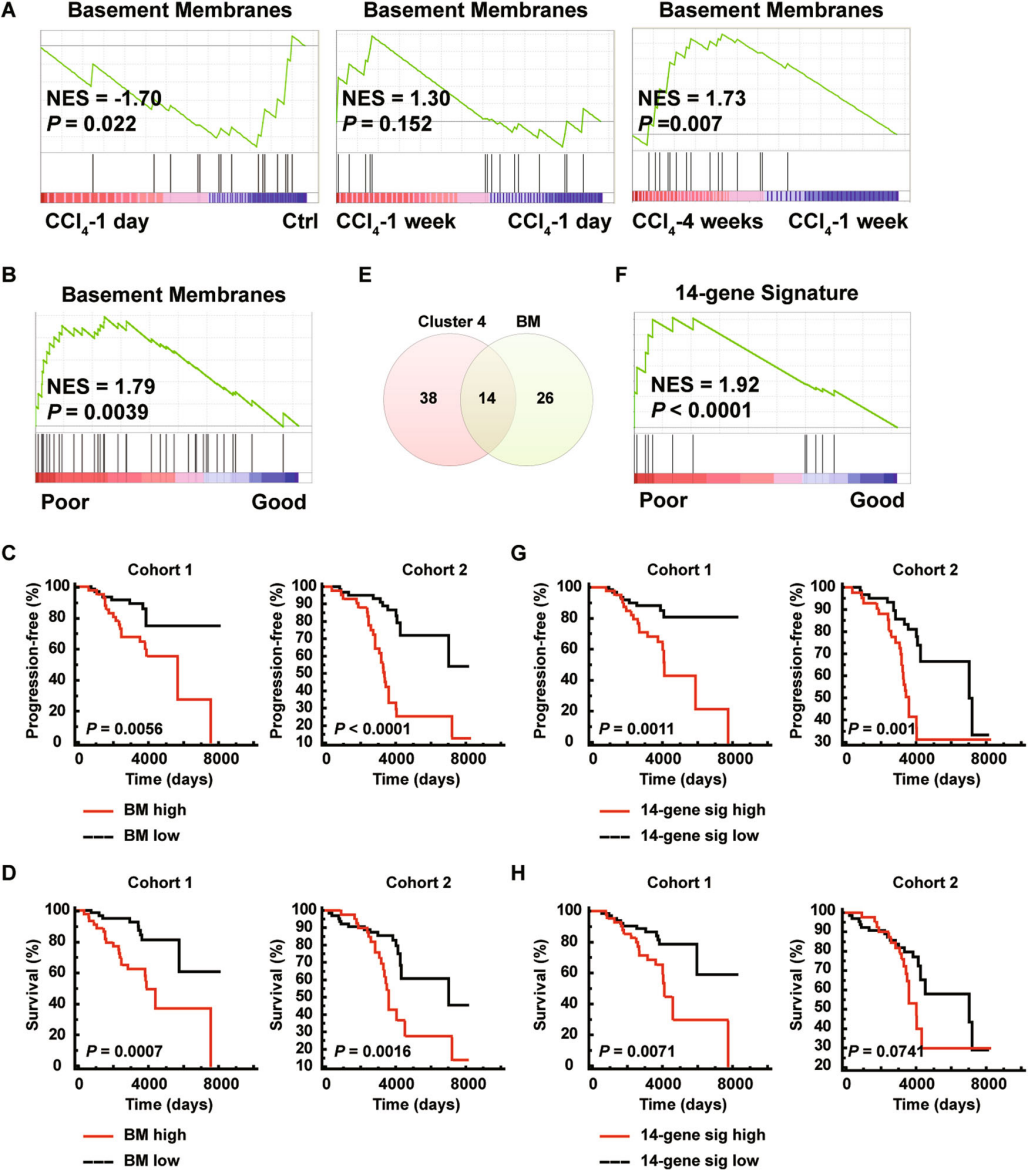
Basement membrane proteins are dynamically deposited in CCl_4_-induced liver fibrosis, and predict disease progression and survival of early-stage liver cirrhosis patients. **A** GSEA analyses comparing enrichment of core basement membrane signature in hepatic matrices across four liver fibrogenesis stages. **B** GSEA analyses comparing enrichment of core basement membrane signature between good prognosis and poor prognosis Child-Pugh Class A early-stage liver cirrhosis biopsies. **C**, **D** Probabilities of disease progression from Child-Pugh Class A to Class B or C (**C**) and survival (**D**) of early-stage cirrhosis patients according to the expression level of the core basement membrane signature. **E** Venn diagram depicting overlap of cluster-4 signature and basement membrane signature. **F** GSEA analyses comparing enrichment of 14-gene signature between good prognosis and poor prognosis Child-Pugh Class A early-stage liver cirrhosis biopsies. **G**, **H** Probabilities of disease progression from Child-Pugh Class A to Class B or C (**G**) and survival (**H**) of early-stage cirrhosis patients according to the expression level of the 14-gene signature.

BMs are specialized ECMs underneath epithelial cells, endothelial cells, and smooth muscle cells^31,32^. Type IV collagens, laminins, perlecan, nidogens, type XVIII collagen and type XV collagen are the core BM components^10,33^. Consistent to damaged tissue architecture, core BM components^10^ were downregulated after 1-day CCl_4_ treatment (Fig. 3A). BMs were partially restored after 1-week and fully restored after 4-week CCl_4_ treatment (Fig. 3A). BMs are highly upregulated in liver fibrosis and cirrhosis^34,35^. Core BM components were enriched in the poor prognosis Child-Pugh Class A liver cirrhosis patient group (Fig. 3B and S5B). High core BM protein expression correlated to shorter disease progression-free (Cohort-1: HR: 2.97 (1.38–6.42), *P* = 0.0056; Cohort-2: HR: 5.75 (2.94–11.24), *P* < 0.0001) and overall survival (Cohort-1: HR: 3.57 (1.71–7.44), *P* = 0.0007; Cohort-2: HR: 3.07 (1.53–6.17), *P* = 0.0016) (Figs. 3C, D). Ten-year Child-Pugh class progression rates were 32% vs. 11% (Cohort-1) and 67% vs. 13% (Cohort-2) for patients with high or low BM signature (Fig. 3C). Ten-year survival rates were 63% vs. 90% (Cohort-1) and 43% vs. 85% (Cohort-2) for patients with high or low BM signature (Fig. 3D). Clinical variables, e. g. bilirubin level and platelet count, are used to assess the prognosis of liver cirrhosis^4^. Multivariable Cox analyses showed that the basement membrane signature remained significant for the association with progression of Child-Pugh class (HR: 3.19 (1.87–5.42), *P* < 0.0001) and patient death (HR: 2.57 (1.54–4.30), *P* = 0.0003) (Table S5). The presence of both high basement membrane signature and high bilirubin level increased the hazard ratio for progression of Child-Pugh class (HR: 10.71 (4.47–25.64), *P* < 0.0001) and patient death (HR: 6.79 (3.29–14.01), *P* < 0.0001) (Table S6), suggesting that basement membrane signature and bilirubin level is complementary in determining patient prognosis. Indeed, patients with both variables high had the shortest disease progression-free survival (*P* < 0.0001) and overall survival (*P* < 0.0001) (Fig. S6A and S6B). Ten-year Child-Pugh class progression rates were 62 and 4% for patients with high BM signature/high bilirubin level and low BM signature/low bilirubin level, respectively (Fig. S6A). Ten-year survival rates were 40 and 90% for patients with high BM signature/high bilirubin level and low BM signature/low bilirubin level, respectively (Fig. S6B).

Cluster-4 and basement membrane signatures share 14 common proteins, including α chains of type IV, VI, XV and XVIII collagens, laminins, agrin, nidogen and HSPG2 (Fig. 3E, Table S8). Such 14-gene signature is highly enriched in the poor prognosis group of Child-Pugh Class A liver cirrhosis patient samples (Fig. 3F). Univariable Cox analyses indicated that the 14-gene were associated with disease progression (HR: 3.11, *P* < 0.0001) and patient survival (HR: 2.14, *P* = 0.0022) (Table S3). High 14-gene signature expression correlated to shorter disease progression-free (Cohort-1: HR: 3.71 (1.69–8.14), *P* = 0.0011; Cohort-2: HR: 1.57 (1.57–5.82), *P* = 0.001) and overall survival (Cohort-1: HR: 2.79 (1.32–5.88), *P* = 0.0071; Cohort-2: HR: 1.87 (0.94–3.71), *P* = 0.074) (Figs. 3G, H). Ten-year Child-Pugh class progression rates were 32% vs. 12% (Cohort-1) and 58% vs. 19% (Cohort-2) for patients with high or low 14-gene signature (Fig. 3C). Ten-year survival rates were 65% vs. 87% (Cohort-1) and 53% vs. 80% (Cohort-2) for patients with high or low 14-gene signature (Fig. 3D). 14-gene signature was significantly association with progression of Child-Pugh class (HR: 2.83 (1.70–4.70), *P* < 0.0001) and patient death (HR: 2.07 (1.26–3.39), *P* = 0.0042) in multivariable Cox analyses (Table S5). The presence of both 14-gene signature and high bilirubin level increased the risk of disease progression (HR: 6.12 (2.75–13.63), *P* < 0.0001) and patient death (HR: 4.01 (1.97–8.20), *P* = 0.0001) (Table S6). Patients with high 14-gene signature and high bilirubin level had the shortest progression-free survival (*P* < 0.0001) and overall survival (*P* < 0.0001) (Fig. S6C and S6D). Ten-year Child-Pugh class progression rates were 62 and 6% for patients with high 14-gene signature/high bilirubin level and low 14-gene signature/low bilirubin level, respectively (Fig. S6C). Ten-year survival rates were 43 and 88% for patients with high 14-gene signature/high bilirubin level and low 14-gene signature/low bilirubin level, respectively (Fig. S6D).

### Minor type IV collagens are prominent for the development of liver fibrosis

**Fig. 4:**
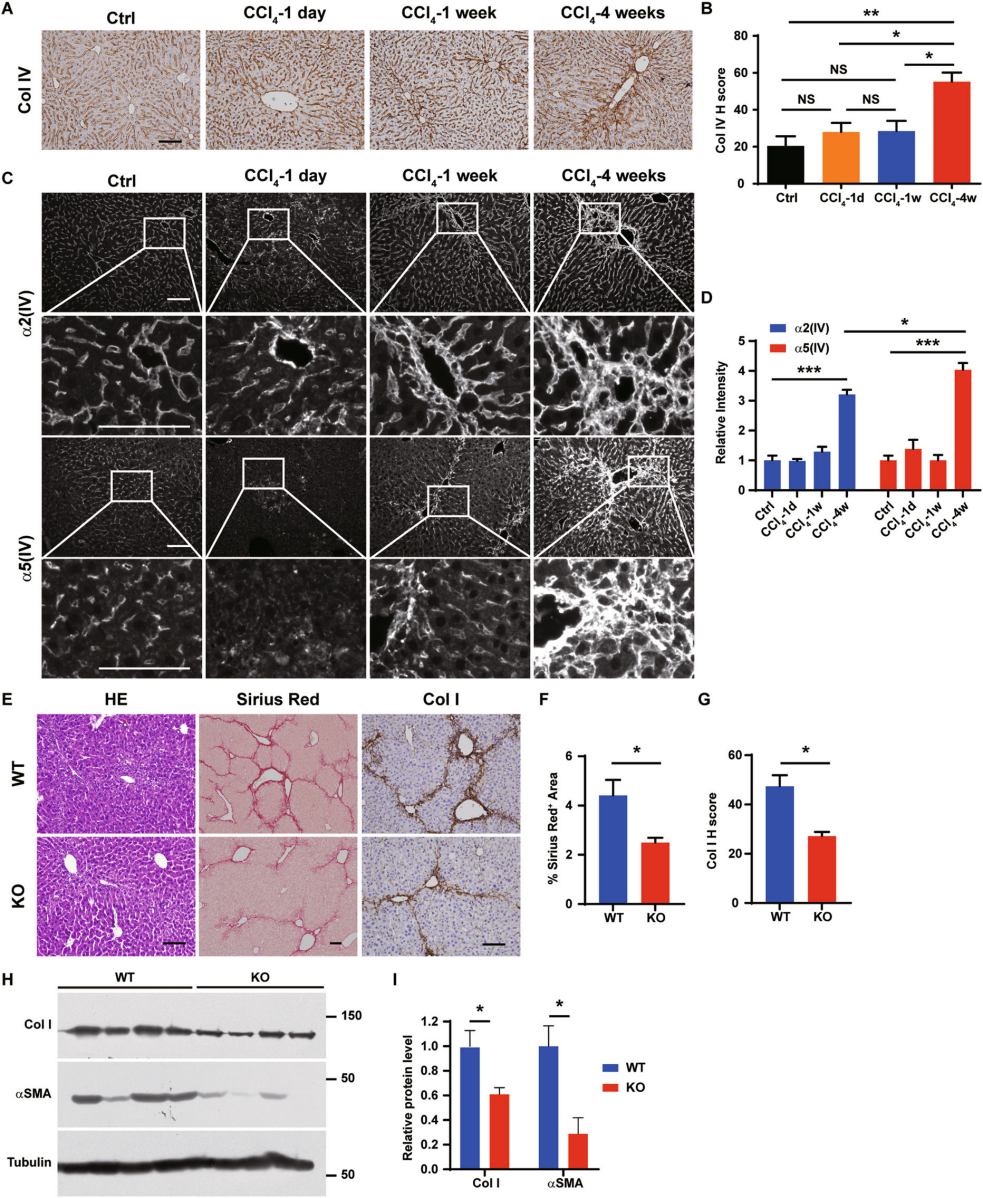
Minor type IV collagen is essential for CCl_4_-induced liver fibrosis. **A**, **B** Immunostaining of type IV collagen on liver sections. Quantification of Col IV is shown in **B**. *N* = 3. Scale bars: 100 μm. **C**, **D** Immunostaining of major type IV collagen α2 chain and minor type IV collagen α5 chain on liver sections. Quantification of α2 chain and α5 chain is shown in **D**. *N* = 3 (1-day and 1-week) or 4 (Control and 4-week). Scale bars: 100 μm. **E**–**H** Wild-type (WT) and *Col4a5* knockout (KO) mice were intraperitoneally injected with CCl_4_ for 4 weeks. **E**–**G** Liver sections are stained with hematoxylin-eosin (HE), Picrosirius Red or immunostained for type I collagen (Col I). Quantification of percentage of Picrosirius Red-positive area and Col I are shown in **F**, **G**. *N* = 3. Scale bars: 100 μm. **H**, **I** Western blot analyses of Col I and αSMA in the livers of WT and KO mice intraperitoneally injected with CCl_4_ for 4 weeks. Quantification is shown in **I**. *N* = 4. Data are presented as mean ± SEM. Statistical analyses were performed with one-way Anova followed by Tukey’s post-hoc analysis or two-tailed unpaired student’s *t* test. *P < 0.05.

Type IV collagens, the core components of BMs essential for the maintenance of BM structure^36,37^, were expressed in the liver and highly upregulated after 4-week CCl_4_ treatment (Figs. 4A, B), consistent to the findings that type IV collagens are accumulated in human liver fibrosis and cirrhosis^34,35^. There are 6 highly homologous type IV collagen α chains (α1(IV)-α6(IV))^36,37^. Type IV collagen α chains form α1α1α2(IV), α3α4α5(IV), and α5α5α6(IV) heterotrimers, in which α1α1α2(IV), known as major type IV collagen, is broadly and abundantly expressed and α3α4α5(IV) and α5α5α6(IV), known as minor type IV collagens, are expressed at low abundance^36,37^. Peptides of type IV collagens identified in the proteomic analyses were conserved between α1(IV) and α5(IV) chains (Table S7). Both major type IV collagen α2(IV) chain and minor type IV collagen α5(IV) chain were localized to the Disse space in the liver (Fig. 4C). Major type IV collagen was unchanged after 1-day and 1-week CCl_4_ treatment, but was accumulated (3.2-fold compared to Oil control) at the fibrotic lesions after 4-week CCl_4_ treatment (Figs. 4C, D and S7). α5(IV) was more substantially upregulated (4-fold compared to Oil control) than α2(IV) after 4-week CCl_4_ treatment (Figs. 4C, D and S7). Despite the total signal intensity of α5(IV) was not changed after 1-day CCl_4_ treatment, α5(IV) was only detected as fragmented dot-like structure, unlike the continuous structures observed in the control livers or after 1-week or 4-week CCl_4_ treatment, indicating disrupted α5(IV) structure after 1-day CCl_4_ treatment (Fig. 4C). Unlike α5(IV), α2(IV) was present as normal continuous structures at all stages during CCl_4_-induced liver fibrosis (Fig. 4C). The dynamic changes of minor type IV collagen expression and structure in CCl_4_-induced liver fibrosis indicated that minor type IV collagens may play prominent regulatory roles in hepatic fibrogenesis.

**Fig. 5:**
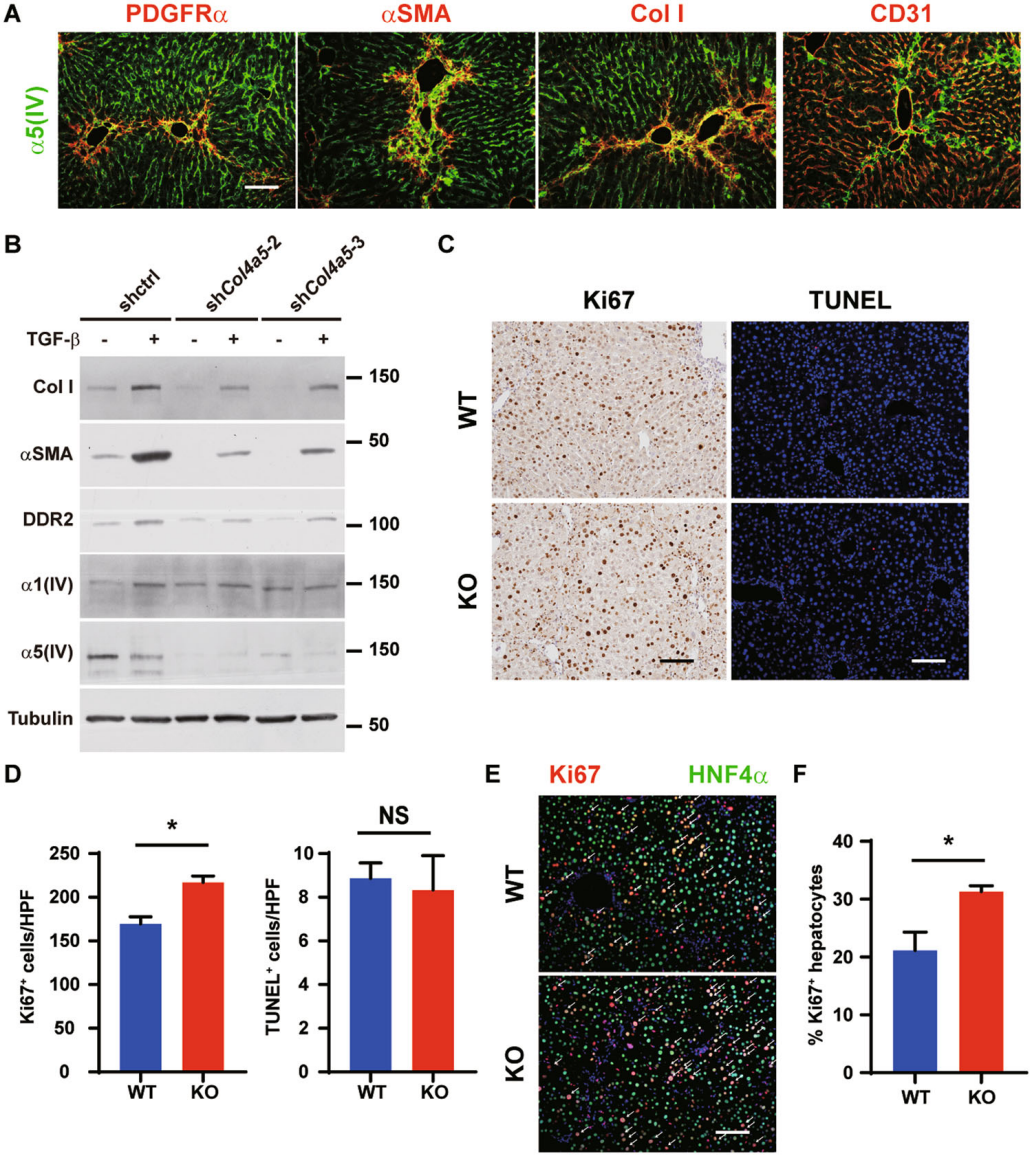
Minor type IV collagen regulates hepatic stellate cell activation and hepatocyte proliferation in CCl_4_-induced liver fibrosis. **A** Liver sections are stained with Col I, αSMA, PDGFRα, CD31 with α5(IV). Scale bars: 100 μm. **B** Western blot analyses of Col I, αSMA, DDR2, α5(IV) and α1(IV) in α5(IV) knock-down rat hepatic stellate cell line CSFC-8B treated with or without 1 ng/mL TGF-β for 48 h. **C**, **D** Liver sections are immunostained for Ki67 or TUNEL. Quantification of Ki67 or TUNEL is shown in **D**. *N* = 3. **E**, **F** Liver sections are immunostained for Ki67 and HNF4α (**E**). Ki67^+^HNF4α^+^ hepatocytes are marked by white arrows. Percentage of Ki67^+^ cells in HNF4α^+^ hepatocytes is shown in **F**. *N* = 3. Scale bars: 100 μm. Data are presented as mean ± SEM. Statistical analyses were performed with two-tailed unpaired student’s *t* test. *P < 0.05. NS: Not significant.

To investigate the functions of minor type IV collagens in liver fibrosis, mice deficient of α5(IV)^38^ were challenged with CCl_4_ (Fig. 4E–H, S8). Significantly less severe liver fibrosis developed in the KO mice after 4-week CCl_4_ treatment (Fig. 4E–I). α5(IV) is expressed in the endothelial cells^38^. α5(IV) partially colocalized with endothelial cell marker CD31 in the liver (Fig. 5A). Portal angiogenesis was reported to attenuate fibrogenesis, whereas sinusoid capillarization was believed to promote fibrogenesis^39^. Despite knockdown of α5(IV) impaired endothelial cell proliferation in vitro (Fig. S8A and S8B)^38^, α5(IV) deficiency did not affect either portal angiogenesis or sinusoid capillarization (Fig. S8C–S8F).

### Minor type IV collagens regulate hepatic stellate cell activation and hepatocyte proliferation

Hepatic stellate cells (HSCs) are the main producers of ECM in hepatocytic damage-induced liver fibrosis^40,41^. After 4-week CCl_4_ treatment, α5(IV) partially colocalized with the HSC marker PDGFRα (Fig. 5A). At the fibrotic lesions, α5(IV) was expressed in majority of the αSMA and type I collagen (Col I)-positive activated HSCs (Fig. 5A). Knockdown of α5(IV) in CSFC-8B HSC cells significantly reduced basal and TGF-β-induced expression of type I collagen and αSMA (Fig. 5B). α5(IV) deficiency in lung cancer cells resulted in decreased expression of non-integrin collagen receptor discoidin domain receptor-1 (DDR1), and α5(IV) regulates cell proliferation via DDR1^38^. DDR1 is mainly expressed in epithelial cells, and its family member DDR2 is expressed in mesenchymal cells^42^. While DDR1 is not expressed in HSCs, DDR2 expression is induced in HSCs during liver injury^43^. DDR2 is induced in activated HSCs and is required for HSC activation. TGF-β treatment increased DDR2 expression in CSFC-8B cells (Fig. 5B). DDR2 knockdown reduced expression of type I collagen and αSMA in CSFC-8B cells (Fig. S9A). DDR2 expression was significantly reduced in α5(IV)-deficient livers (Fig. S9B and S9C). Knockdown of α5(IV) in CSFC-8B cells significantly reduced DDR2 expression (Fig. 5B), suggesting α5(IV) may regulate HSC activation and liver fibrosis through DDR2.

Damage-induced liver fibrosis is a consequence of imbalanced tissue damage and tissue repair. α5(IV) deficiency may promote functional liver regeneration upon CCl_4_ challenge. α5(IV) deficiency did not affect hepatocyte cell death after 4-week CCl_4_ treatment (Figs. 5C, D). However, α5(IV) deficiency significantly increased the numbers of Ki67-positive proliferating cells after 4-week CCl_4_ treatment (Figs. 5C, D). Consistently, increased PCNA and cyclin D1 expression was detected in α5(IV)-deficient livers after 4-week CCl_4_ treatment (Fig. S10A and S10B). Upon injury, hepatocytes proliferate to recover from damage. Significantly more proliferating HNF4α-positive hepatocytes were evident in α5(IV)-deficient livers after 4-week CCl_4_ treatment (Figs. 5E, F). Activation of ERK1/2 and AKT signaling pathways play a major role in liver regeneration^44,45^. Consistent to higher percentage of proliferating hepatocytes, phosphorylation of ERK1/2 and AKT was significantly increased in α5(IV)-deficient livers after 4-week CCl_4_ treatment (Fig. S10C and S10D).

## Discussion

Liver cirrhosis remains major health problem^1,2^. With the progress on diagnosis of asymptomatic early-stage liver cirrhosis, prognostic biomarkers are becoming even more urgent medical needs to identify cirrhotic patients at high risk developing advanced stage disease, and to guide the follow-up therapy after diagnosis. In this study, we analyzed dynamic changes of hepatic matrisome in the development of liver fibrosis, and identified matrisomal protein signatures as potential prognostic biomarkers for early-stage liver cirrhosis, in which minor type IV collagens play prominent roles in the development of liver fibrosis.

Serum biomarkers are currently available for non-invasive evaluation of liver fibrosis in chronic liver disease, e. g. Fibrotest®, AST to Platelet Ratio (APRI), FibroSpectII®, Enhanced Liver Fibrosis score® (ELF)^3^. Liver stiffness measurement with transient elastography and other liver elasticity-based imaging techniques offer another non-invasive approach for early diagnosis of liver fibrosis and cirrhosis^3^. Despite the success in diagnosis of liver cirrhosis, in particular asymptomatic early-stage cirrhosis, these diagnostic methods are not robust to classify early-stage cirrhosis patients into prognostic subgroups^3^. Age, sex, platelet count, and aspartate aminotransferase/alanine aminotransferase ratio were identified independently associated with mortality of chronic HCV infection and compensated advanced liver fibrosis^46^. Child-Pugh score, consisting of total bilirubin level, serum albumin level, prothrombin time, the degree of ascites, and the grade of hepatic encephalopathy, is used to assess the prognosis of liver cirrhosis^4^. In addition to clinical variables, gene signatures have been used to assess the clinical outcome of complex diseases. A prognostic 186-gene signature was reported to predict disease progression to advanced stage cirrhosis, patient survival and hepatocellular carcinoma development of HCV-related Child-Pugh Class A early-stage cirrhosis patients^5,47^.

Liver fibrosis is part of the general wound healing response to many causes of chronic injury^7,8^. Liver fibrosis and cirrhosis are characterized by aberrant deposition of ECM proteins. Individual ECM components have been proposed as diagnostic and prognostic biomarkers for liver fibrosis and cirrhosis. Matrisomal proteins are part of the 186-gene signature^5,47^, including collagens, glycoproteins, proteases, and secreted factors. Elastin is a key glycoprotein that provides elasticity to tissues. Although elastin is weakly expressed in normal liver, elastin is abundantly deposited at late stages of liver fibrosis^48^. Hepatic elastin content is predictive of adverse outcome in advanced fibrotic liver disease^49^. Elastin imaging enables staging and longitudinal monitoring of kidney fibrosis therapeutic interventions^50^. ECM remodeling is highly dynamic and complex in tissue homeostasis and diseases. Proteomic analyses of matrisome composition of normal and diseased tissues provide insights into mechanisms regulating fibrotic diseases^10–17^. In this study, we identified cluster-3 and cluster-4 matrisomal proteins as potential prognostic biomarkers for early-stage cirrhosis. Cluster-3 matrisomal proteins (24 genes) are responsible for fibrinolysis and homeostatic remodeling. Cluster-3 signature is associated with good prognosis of HCV-related early-stage cirrhosis patients (Fig. 2, Table S3 and S4). Cluster-4 ECM signature (52 genes) includes core ECM proteins previously reported to regulate liver fibrogenesis, e.g., collagens, elastin and small leucine-rich proteoglycans^51^. Cluster-4 signature predicts poor prognosis of HCV-related early-stage cirrhosis patients (Fig. 2, Table S3 and S5). In particular, patients with low cluster-3 signature and high cluster-4 signature had significantly shorter disease progression-free survival and overall survival (Fig. 2), highlighting the importance of balanced tissue repair and fibrogenesis in the development of cirrhosis.

Besides cluster-3 and cluster-4 signatures, BM signature is potent in predicting early-stage liver cirrhosis disease progression and patient survival (Fig. 3). At the stage when fibrotic lesions were prominent, components of BMs^33^ were enriched in the hepatic matrix, including type IV, XV, XVIII collagens, laminin 521, nidogens, and heperan sulfate proteoglycans perlecan and agrin (Table S2). A 14-gene signature, present in both cluster-4 and basement membrane signatures, is potent in predicting disease progression and patient survival (Fig. 3). Clinical variables, e. g. bilirubin level and platelet count, are used to assess the prognosis of liver cirrhosis^4^. Interestingly, patients with high basement membrane signature/14-gene signature and high bilirubin level had the shortest disease progression-free survival and overall survival, while patients with low basement membrane signature/14-gene signature and low bilirubin level had the best prognosis (Fig. S7).

Type IV collagens, core components of BMs, are deposited at the Disse space^35^ and upregulated in fibrotic livers^34^. Type IV collagen networks are essential in maintaining BM structure^36,37^. Major and minor type IV collagens were both extensively deposited to fibrotic lesions (Fig. 3F). Minor type IV collagen expression and structure were dynamically changed during the development of liver fibrosis (Fig. 4 and S7), suggesting minor type IV collagens may play prominent roles in regulating liver fibrosis. Minor type IV collagens are highly expressed in activated hepatic stellate cells. Deficiency of minor type IV collagens resulted in reduced activation of hepatic stellate cells and much reduced liver fibrosis (Figs. 4 and 5). On the other hand, minor type IV collagens regulated hepatocyte proliferation and the wound healing process (Fig. 5). The essential roles of type IV collagens in the development of liver fibrosis indicate that the prognostic value of basement membrane is closely related to its functions regulating liver cirrhosis development. Minor type IV collagens may regulate HSC activation via non-integrin collagen receptor DDR2, which was previously reported to be induced in HSCs during liver injury and mediates HSC activation^43^. Type IV collagens bind soluble glycoproteins, proteoglycans and growth factors in a tissue-specific manner to establish fully functional BMs^52^. Hepatocyte growth factor (HGF) is an HSC-derived paracrine factor regulating liver cancer cell proliferation via its receptor c-Met and downstream MAPK and Akt pathways^53^. It warrants further investigation whether minor type IV collagens regulated hepatocyte proliferation through HGF and possibly other growth factors.

In summary, unique matrisomal proteins were identified for tissue damage/inflammation, transitional ECM remodeling and fibrogenesis. Such matrisomal protein signatures, in particular basement membrane signature, are predictive of early-stage cirrhosis progression and patient survival, and may help development of biomarkers for the prediction of clinical outcomes of liver cirrhosis patients at very early stage.

## Materials and methods

### Liver injury and fibrosis models

Male C57BL/6 mice were purchased from SLAC (Shanghai). *Col4a5* knockout mice were maintained in C57/BL6 background as described^38^. 8-week-old wild-type or *Col4a5* knockout mice were randomly divided to four groups, and were intraperitoneally injected with 1 μl/g body weight CCl_4_ (dissolved in olive oil at a ratio of 1:4) (Sigma-Aldrich) or olive oil twice a week for 1 day, 1 week or 4 weeks to induce liver injury and liver fibrosis as described^23^. The mice were euthanized by inhaling CO_2_ at 1 day, 1 week and 4 weeks after initial CCl_4_ injection. Livers were fixed in 4% paraformaldehyde and processed for paraffin embedding, or were snap frozen in liquid nitrogen and stored at −80 °C. No blinding was performed.

### Decellularization and ECM protein enrichment

Three biological repeats were obtained for each represented stage. Decellularization and ECM protein enrichment were performed as described with modifications^54^. ~ 50 mg liver was homogenated in 2 mL extraction buffer (10 mM Tris-HCl, pH 7.5, 150 mM NaCl, 1% Triton X-100, 10 mM EDTA, 1× Roche protease inhibitors), and incubated for 16 h to solubilize cellular proteins. Pellets from centrifuged tissue homogenates were resuspended in extraction buffer and incubated for another 16 h. Samples were then centrifuged and the remaining pellet was washed once with 10 mM Tris-HCl, pH 7.5, 150 mM NaCl, and incubated overnight in deoxyribonuclease buffer (100 ug/ml deoxyribonuclease I in 10 mM Tris-HCl, pH 7.5, 2.5 mM MgCl_2_, 0.5 mM CaCl_2_) to degrade DNA. The samples were centrifuged, and the final pellet was washed with PBS three times to yield the enriched ECM fraction. All steps were carried out at 4 °C to minimize proteolysis.

### LC-MS/MS for ECM proteome analysis

Enriched ECM proteins were digested into peptides as previously reported^55^ with slight modification. In brief, ~5 mg dry weight ECM-enriched pellets were resuspended in 8 M urea with a final concentration of 40 mM DTT at 1400 rpm for 2 h at 37 °C. Protein samples were cooled to room temperature and IAA was added to a final concentration of 100 mM. Akylation reactions last 30 min in darkness at room temperature (RT). Urea was diluted to 2 M with 100 mM ammonium bicarbonate, pH 8.0. Deglycosylation with 1000 U PNGase F per sample was preceded for 2 h at 37 °C with continuous agitation at 1400 rpm. 1 μg endoproteinase Lys-C was then added to each sample and incubated at 1400 rpm for 2 h at 37 °C. The first aliquot of 3 μg trypsin was added to each sample, followed by overnight incubation at 37 °C with continuous agitation at 1400 rpm. A second aliquot of 3 μg trypsin was then added to each sample and incubated at 1400 rpm for an additional 2 h at 37 °C. ~1 μl freshly prepared 50% TFA per sample was added to inactivate trypsin after the protein digestion. After centrifugation at 16,000×*g* for 5 min at RT, all the supernatants were collected into clean tubes, desalted by StageTips^56^. Peptide concentrations were measured assuming a mean tryptophan content of 1.3%^57^.

The purified peptide samples were analyzed on the Thermo Scientific™ Q Exactive™ HF-X hybrid quadrupole-Orbitrap mass spectrometer coupled to HPLC via a nanoelectrospray ion source. The LC-MS/MS method was similar to the previous reports^58,59^ with minor modifications. Briefly, each peptide sample was loaded directly onto a spray analytical column (75 μm inner diameter, ~150 mm length) packed in-house with ReproSil-Pur C18-AQ, 3 μm resin (Dr. Maisch, GmbH) using a 60 min gradient B (0.1% formic acid in acetonitrile) from 5 to 90% at a flow rate of approximate 300 nL/min after split. The analytical column was previously equilibrated to 95% Mobile Phase A (0.1% formic acid in water) and 5% Mobile Phase B (0.1% formic acid in acetonitrile) and maintained at a constant column flow of 300 nL/min. The gradient profile (min: %B) was as shown in the following: 0:5, 2:8, 42:23, 50:40, 52:90, 60:90. A lock-mass *m*/*z* 445.12003 was used for internal calibration. Electrospray voltage (2.5 kV) was applied between the analytical column and electrospray emitter. The capillary temperature was set at 300 °C. Orbitrap precursor spectra were collected from 300–1500 m/z for 60 min at a resolution of 120 K (AGC target 3 × 10^6^, maximum ion time of 20 msec) along with the top 25 data dependent Orbitrap HCD MS/MS spectra at a resolution of 15 K (AGC target 1 × 10^5^, maximum ion time of 25msec). Masses selected for MS/MS were isolated at a width of 1.2 m/z and fragmented using a normalized collision energy of 28%. Exclude isotopes was set to ‘on’, and charge state screening was enabled to reject unassigned, 1+, 7+, 8+, and >8+ ions with a dynamic exclusion time of 30 s to discriminate against previously analyzed ions between ±12 ppm.

### Quantification of ECM proteomics data, protein annotation and functional analysis

Considering various intracellular and extracellular post-translational modifications (PTMs) involved in the generation and maturation of ECM proteins (especially for collagens)^60,61^, dominant PTMs were firstly scanned in the MS data using an open search engine pFind (V3.1.5, 64 bit for windows, released on Jan. 24, 2019)^62^. Carbamidomethyl (C), oxidation (M), deamidated (N), hydroxyproline, and hydroxylysine were ranked the 1st, 2nd, 3rd, 8th, and 28th place with 9.56%, 6.62%, 5.60%, 0.88% and 0.09% of all identified spectra, respectively. All acquired spectra were then searched by MaxQuant version 1.6.2.10 against the UniProt Mouse protein database (07/2017 download, 82051 sequences) with contaminant sequences and decoy sequences for label-free peptide quantification^63,64^. Main search parameters used (Carbamidomethyl (C), oxidation (M) and deamidated (N)) were specified as variable modifications. The precursor mass tolerance for protein identification on MS was 4.5 ppm, and the product ion tolerance for MS/MS was 7 ppm. Full cleavage by trypsin was used, with up to two missed cleavages allowed. A 1% FDR at the PSM, peptide and protein level, peptides with a minimum length of seven amino acids. Label-free quantification was performed with normalized protein intensity using a minimum ratio count of 2. As for proteins with hydroxyproline and hydroxylysine, carbamidomethyl (C), oxidation (M), hydroxyproline and hydroxylysine were specified as variable modifications. Other parameters were the same as the above description and removed all the scans identified in previous searching by manual check. Protein classification was performed with in silico Matrisome annotation tool (mouse Matrisome)^10^. R version 3.6.0 (http://www.R-project.org/) was used to perform mass-spec data normalization, statistical analysis, PCA (principal component analysis), HCA (hierarchical cluster analysis), and C-Means Clustering. After averaging the three biological replicates for each time points, the data matrix was standardized using R package Mfuzz. 152 matrisomal proteins were classified into 4 clusters with the degree of fuzzification *m* = 2 based on fuzzy c-means (FCM) algorithm. This parameter combination gave a clear temporal classification for the four time points, without overlapping clusters or empty clusters. The maximum membership score of the given protein was considered for partitioning it to the corresponding cluster (Table S2). Kruskal–Willis test was used to select differentially expressed proteins. Gene ontology (GO) analysis was performed with the DAVID online tool. Top GO categories were selected according to the *P* value after Benjamini-Hochberg correction. Gene set enrichment analyses (GSEA) were performed on matrisome gene signatures^10^ obtained from the MSigDB database v5.0 (March 2015 release)^27^, or from the clusters identified in the mass-spec analysis in this study. Gene signatures used in the GSEA analysis are provided in Table S8. Statistical significance of GSEA analysis was assessed by comparing the enrichment score to enrichment results generated from 1,000 random permutations of the gene set to obtain *P* values (nominal *P* value).

### Cell lines

CSFC-8B hepatic stellate cells (ATCC) were maintained in RPMI 1640 (Hyclone) supplemented with 10% FBS (Biochrom). 293T and EA.hy926 cells (ATCC) were cultured in DMEM (GIBCO) with 10% FBS. All cell lines were routinely tested for mycoplasma contamination. shRNAs targeting *Col4a5* were cloned into pLKO.1-puro lenti-viral vector (Addgene). The target sequences are: 5′-CAACAAGATGAAGAGCACCAAC-3′ (sh*Scram*), 5′-GGGTGATGATGGAATTCCA-3′ (sh*COL4A5*–10), 5′-GCCAGAGCAAAGTCTCTATTA-3′ (sh*COL4A5*–16), 5′-GCTCCTGTTTGGAAGAATTTC-3′ (Rat sh*Col4a5*–2), 5′-GCAATGGACTCCCAGGCTTTG-3′ (Rat sh*Col4a5*–3). After viral infection, cells were selected with puromycin to generate stable cell lines. At least two batches of stable cell lines were generated for each experiment. Experiments were performed in triplicates and repeated at least twice. Cell Counting Kit-8 (CCK8) and 5-ethynyl-2′-deoxyuridine (EdU) incorporation assays were performed according to manufacturer’s protocol.

### Histological and immunohistochemical staining

Paraffin-embedded liver tissues were sectioned and stained with hematoxylin and eosin (HE) or Picrosirius Red (Sigma) for gross histology and fibrosis assessment. The immunohistochemical staining was performed using Avidin-Biotin Complex (ABC) method as previously described^65^. Briefly, deparaffined and rehydrated 5 μm liver sections were subjected to heat-induced epitope retrieval with 10 mM Citrate at pH 2.0 (for α2(IV) and α5(IV)) or at pH 6.0. Sections were incubated with diluted primary antibodies at 4 °C overnight, followed by incubation with biotinylated secondary antibodies and horseradish peroxidase-conjugated ABC complex. The primary antibodies used are listed in Table S9. Signals were developed with DAB chromogen and counterstained with hematoxylin. For multiplex immunofluorescent staining, Opal^TM^ fluorophores (PerkinElmer) were used to visualized the signal. Primary and secondary antibodies in previous round immunostaining were stripped by microwave treatment before staining with a second primary antibody. Sections were treated with TrueBlack® Lipofuscin Autofluorescence Quencher (Biotium) before DAPI counterstaining and mounting. Sections were viewed under microscope BX53 with an UPlanSAPO ×20 objective/0.75 (OLYMPUS, Inc.). Images were captured with a digital camera (IX-SPT; OLYMPUS, Inc.) and Digital Acquire software (DPController; OLYMPUS, Inc.). Automated scoring and quantitative evaluation of DAB stained IHC images are performed using open source IHC Profiler Plugin^66^ in ImageJ software. H-score was calculated by adding the percentage of high positive (×3), positive (×2), and low positive cells (×1) as described^67^. H-score has a possible range of 0–300. Quantification of picrosirius red-positive area, integrated fluorescent density, and numbers of Ki67-positive or TUNEL-positive cells are performed using ImageJ software. Percentage of Ki67-positive cells in HNF4α-positive hepatocytes are analyzed with InForm® software (PerkinElmer). The number of CD31-positive vessels surrounding the portal area and the CD31-positive capillarization of liver sinusoids were manually counted as described^39^. All quantification was performed on pictures taken from at least 5 randomly selected 20× fields per mouse. Three to four mice per treatment group were analyzed.

### Western blot and quantitative RT-PCR analysis

Livers were homogenated in SDS sample buffer or Trizol reagents (Invitrogen). Western blot and quantitative RT-PCR analyses were performed as previously described^68^. The primary antibodies and primers used are listed in Tables S9 and S10, respectively. Gene expression levels were normalized to *Actin*. All experiments were performed at least twice.

### Early-stage liver cirrhosis patient disease progression and survival analyses

Previously published Child-Pugh Class A early-stage liver cirrhosis microarray data (GSE15654)^5^ is used to assess the prognostic potential of matrisome cluster signatures. To avoid potential heterogeneity among the samples, 216 samples were randomly split into two data sets with similar size (107 and 109 cases). The two cohorts have similar clinicopathologic characteristics as assessed by chi-square test (Table S4). Gene set enrichment scores of each ECM signature were calculated for each sample using Gene Set Variation Analysis (GSVA)^69^. Survival analysis was performed using the uni-variable and multi-variable Cox regression and Kaplan–Meier (Log-Rank test) method to evaluate the correlation of the ECM signature GSVA score with disease progression and overall survival in these two data sets.

### Statistical analysis

Variance similarity between the groups is being statistically compared. Data meet the assumptions of the tests. No data were excluded from the analysis. Sample size was based on previous research experience. Sample numbers were indicated in figure legends. Statistical significance between conditions was assessed by unpaired two-tailed Student’s *t*-tests or one-way Anova followed by Tukey’s post-hoc analysis using GraphPad Prism. All error bars represent SEM.

## Supplementary information

Supplementary figures

Supplementary tables
