# Supplementary material for: Dynamically remodeled hepatic extracellular matrix predicts prognosis of early-stage cirrhosis
Source: Cell Death Dis. 2021 Feb 8;12(2):163. doi: 10.1038/s41419-021-03443-y (PMC7870969; doi:10.1038/s41419-021-03443-y)
Supplement: Supplementary file 1 — Supplementary figures [file 41419_2021_3443_MOESM1_ESM.docx]

**Dynamically remodeled hepatic extracellular matrix predicts prognosis of early-stage cirrhosis**

Yuexin Wu^1,2^, Yuyan Cao^1,2^, Keren Xu^2,3^, Yue Zhu^1,2^, Yuemei Qiao^1^, Yanjun Wu^1^, Jianfeng Chen^1,4^, Chen Li^5,3,^*, Rong Zeng^3,6,7,^*, Gaoxiang Ge^1,4,^*

**Supplementary Materials**

The supplementary materials contain 10 supplementary figures and 10 supplementary tables.

**Supplementary figures**

**
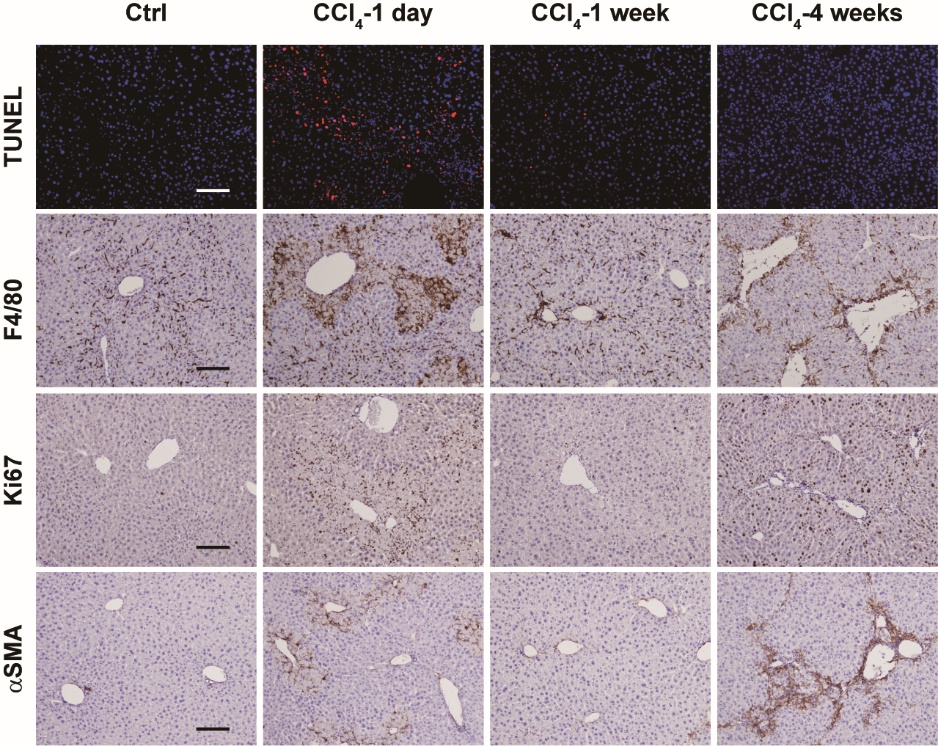
**

**Fig. S1 Hepatic histology in CCl_4_-induced liver fibrosis model.**

Microscopic views of livers from mice intraperitoneally injected with CCl_4_ for 1 day, 1 week or 4 weeks or olive oil for 4 weeks. Liver sections were immunostained for TUNEL, F4/80, Ki67 and α-smooth muscle actin (αSMA). Scale bars: 100 μm.


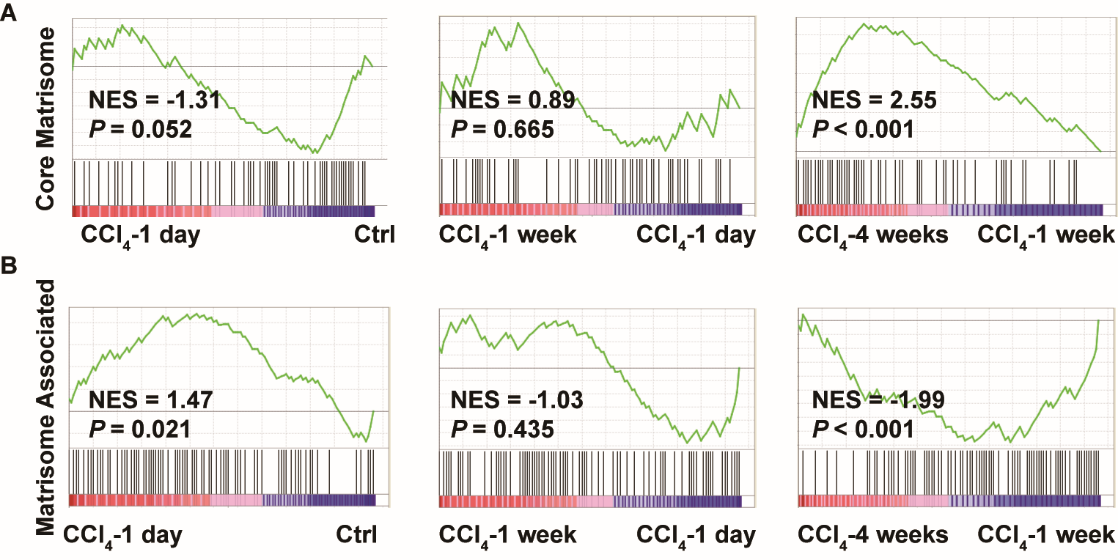


**Fig. S2 GSEA analyses comparing enrichment of core matrisome (A) and matrisome associated (B) signatures in hepatic matrices across four liver fibrogenesis stages.**

**
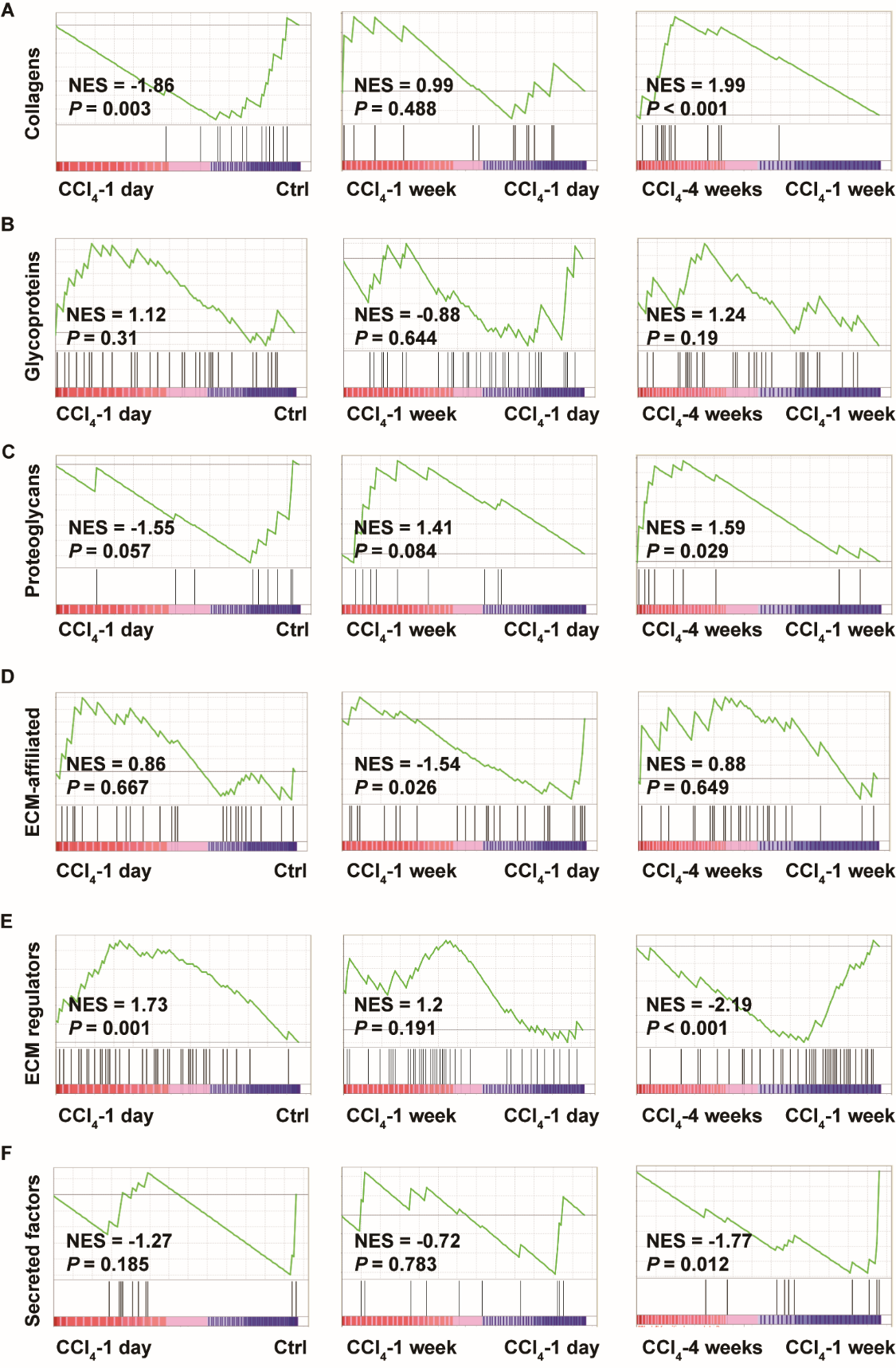
**

**Fig. S3 GSEA analyses comparing enrichment of collagens (A), glycoproteins (B), proteoglycans (C), ECM-affiliated (D), ECM regulators (E) and secreted factors (F) signatures in hepatic matrices across four liver fibrogenesis stages.**

**

Fig. S4 Expression of matrisome in CCl_4_-induced liver fibrosis.**

RT-PCR of *S100a8*, *S100a9*, *Fga*, *Fgb*, *Fgg*, *Col1a1*, *Col5a1*, *Lama5*, *Lamb2*, and *Postn* at different stages of CCl_4_-induced liver fibrosis. Data are presented as mean ± SEM.

**
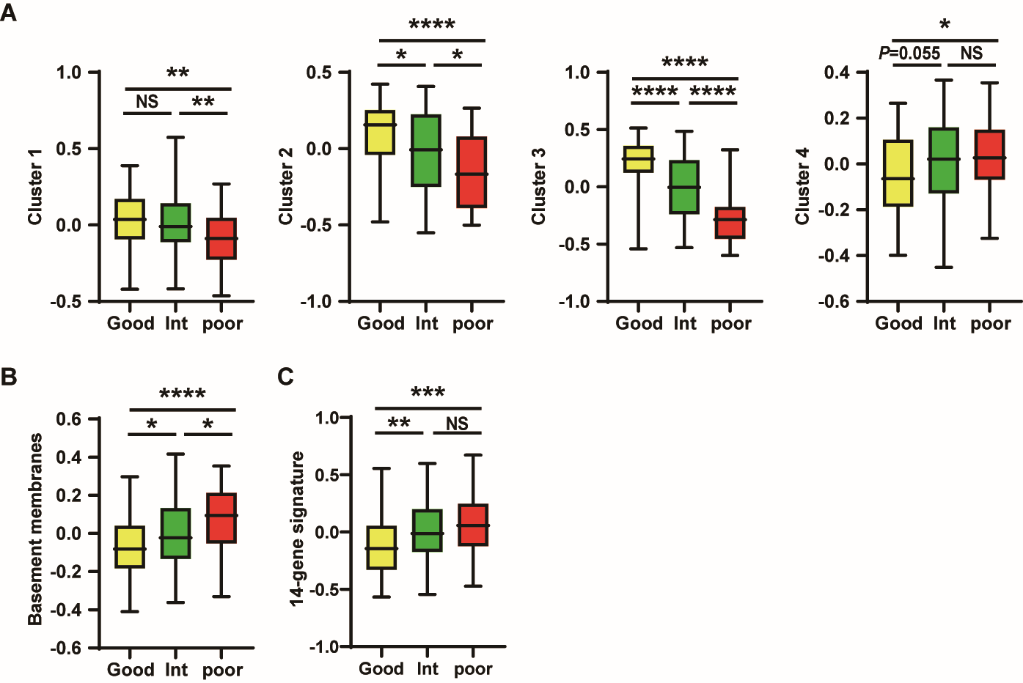
**

**Fig. S5 Box plots of gene set enrichment scores of ECM clusters (A), basement membrane signature (B) and 14-gene signature (C) in good, intermediate and poor prognosis groups in early-stage liver cirrhosis microarray data (GSE15654).**

**
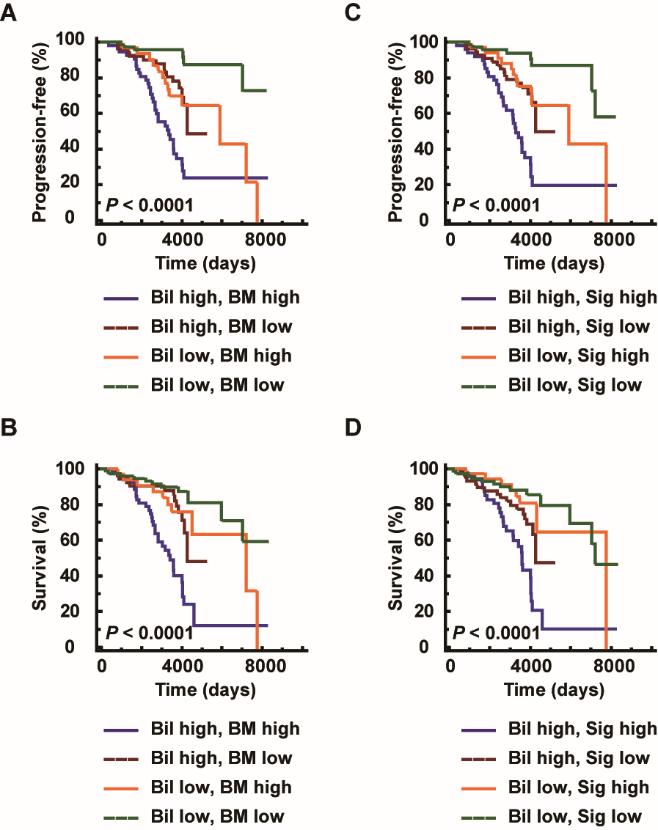
**

**Fig. S6 Composite effect of ECM signatures and bilirubin level on the prognosis of early-stage liver cirrhosis patients.**

Probabilities of disease progression from Child-Pugh class A to class B or C (A and C) and survival (B and D) of early-stage cirrhosis patients according to the expression level of the basement membrane signature (A and B) or 14-gene signature (C and D) with bilirubin level.





**Fig. S7 Type IV collagens dynamically change in CCl_4_-induced liver fibrosis.**

RT-PCR of *Col4a1*-*Col4a6* at different stages of CCl_4_-induced liver fibrosis. Data are presented as mean ± SEM.


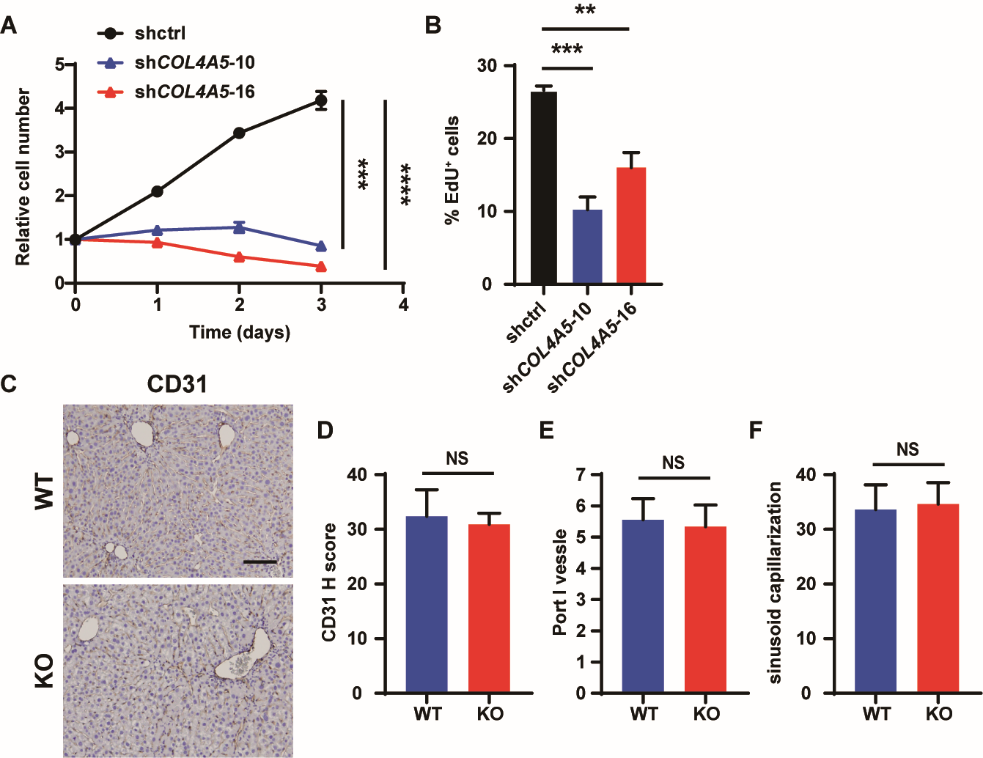


**Fig. S8 Minor type IV collagen deficiency does not affect angiogenesis in CCl_4_-induced liver fibrosis.**

(A and B) Type IV collagen α5 chain was knocked down in EA.hy926 endothelial cells. Cell proliferation was assessed by Cell Counting Kit-8 (CCK-8) (A) and 5-ethynyl-2’-deoxyuridine (EdU) incorporation (B). (C-F) Liver sections are stained with CD31. Quantification of CD31 H-score (D), portal angiogenesis (E) and sinusoid capillarization (F) are shown. N=3. Scale bars: 100 μm.


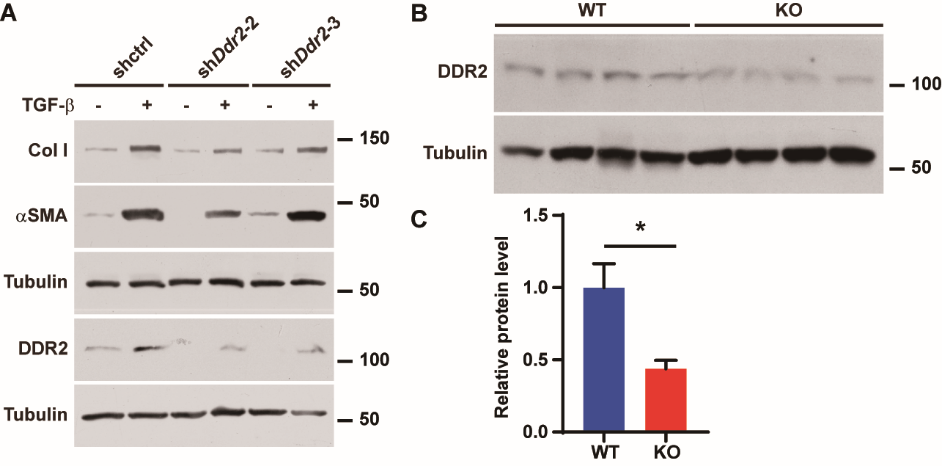


**Fig. 9 DDR2 is required for hepatic stellate cell activation.**

(A) Western blot analyses of Col I, αSMA, DDR2, α5(IV) and α1(IV) in DDR2 knock-down rat hepatic stellate cell line CSFC-8B treated with or without 1ng/mL TGF-β for 48 hours. (B and C) Western blot analyses of DDR2 in the livers of WT and KO mice intraperitoneally injected with CCl_4_ for 4 weeks. Quantification is shown in (C). N=4. Data are presented as mean ± SEM. Statistical analyses were performed with two-tailed unpaired student's *t* test. **P*<0.05.


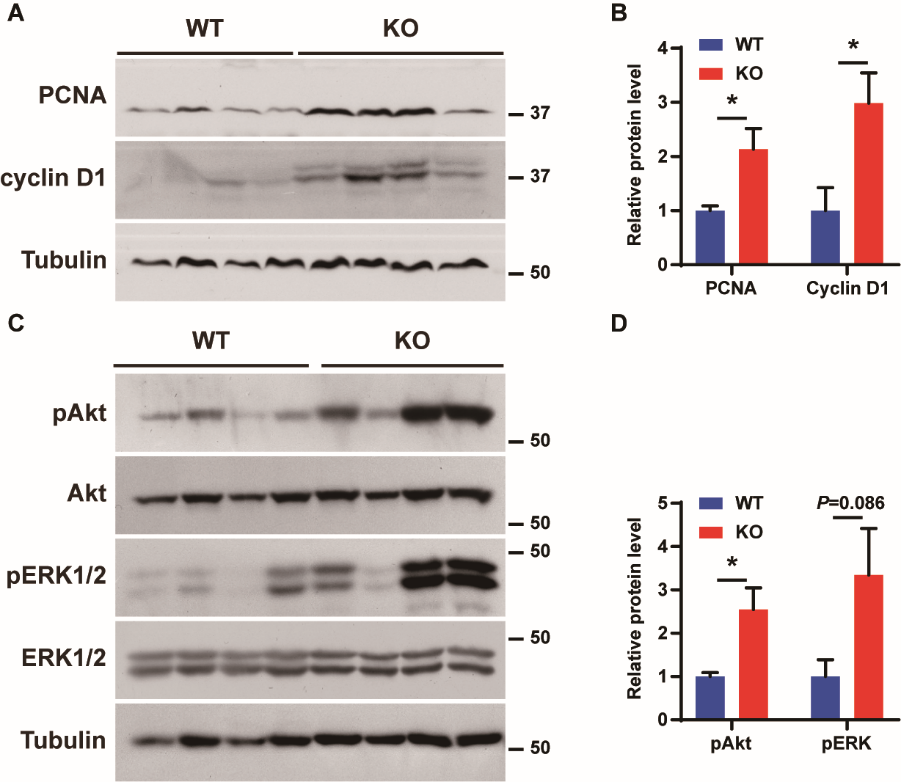


**Fig. 10 Minor type IV collagen regulates hepatocyte proliferation in CCl_4_-induced liver fibrosis.**

(A and B) Western blot analyses of PCNA, cyclin D1in the livers of WT and KO mice intraperitoneally injected with CCl_4_ for 4 weeks. Quantification is shown in (B). (C and D) Western blot analyses of phosphor-ERK1/2 and phosphor-Akt in the livers of WT and KO mice intraperitoneally injected with CCl_4_ for 4 weeks. Quantification is shown in (D). N=4. Data are presented as mean ± SEM. Statistical analyses were performed with two-tailed unpaired student's *t* test. **P*<0.05. NS: Not significant.

**Supplementary tables**

Table S1. Matrisomal proteins identified at each stage of CCl_4_-induced liver fibrosis.

Table S2. Clusters of matrisomal proteins in CCl_4_-induced liver fibrosis.

Table S3. Univariable analysis of ECM signatures with disease progression and death.

Table S4. Comparison of clinicopathologic characteristics of two cohorts of early-stage cirrhosis patients.

Table S5. Multivariable analysis of ECM signatures with disease progression and death.

Table S6.Multivariable analysis of association of composite variable of BM or 14‐gene signature and bilirubin with disease progression and death.

Table S7. Type IV collagen peptides identified in hepatic matrices in CCl_4_-induced liver fibrosis.

Table S8. Gene signatures used in GSEA analysis.

Table S9. Antibodies used in the study.

Table S10. Primers used for qRT-PCR.
